# Central sympatholytics prolong survival in experimental sepsis

**DOI:** 10.1186/cc7709

**Published:** 2009-02-06

**Authors:** Stefan Hofer, Jochen Steppan, Tanja Wagner, Benjamin Funke, Christoph Lichtenstern, Eike Martin, Bernhard M Graf, Angelika Bierhaus, Markus A Weigand

**Affiliations:** 1 Department of Anaesthesiology, University Hospital Heidelberg, INF 110, Heidelberg, 69120, Germany; 2 Department of Anaesthesiology, University of Giessen, Rudolf-Buchheim Strasse 7, Giessen, 35392, Germany; 3 Department of Anaesthesiology, University Hospital Regensburg, Franz-Josef-Strauss-Alle 11, Regensburg, 93042, Germany; 4 Department of Medicine I, University Hospital Heidelberg, INF 410, Heidelberg, 69120, Germany

## Abstract

**Introduction:**

One of the main causes of death in European and US intensive care units is sepsis. It involves a network of pro-inflammatory cytokines such as TNF-α, IL-1β and IL-6. Furthermore, there is an up regulation of transcription factors such as nuclear factor (NF) κB. It has previously been shown that clonidine is able to significantly reduce pro-inflammatory cytokines in surgical patients. We therefore hypothesise that the clinically used central alpha-2 agonist clonidine has the ability to improve survival in experimental sepsis by inhibiting the sympathetic tone and consequently inhibiting the pro-inflammatory cytokine release.

**Methods:**

To investigate this therapeutic potential of clonidine in a prospective randomised laboratory investigation we used a murine model of caecal ligation and puncture (CLP) induced sepsis. Animals receiving pre-emptive injections were treated with either clonidine (5 μg/kg) or dexmedetomidine (40 μg/kg) 12 and 1 hours before the operation, as well as 1, 6 and 12 hours afterwards. Another group of animals only received clonidine (5 μg/kg) 1, 6 and 12 hours after the operation, while the pre-emptive injections were normal saline. The control groups received solvent injections at the respective time points.

**Results:**

Pre-emptive administration of a central sympatholytic significantly reduced mortality (clonidine: p = 0.015; dexmedetomidine: p = 0.029), although postoperative administration of clonidine failed to significantly prolong survival. Furthermore pre-emptive administration of clonidine significantly attenuated the cytokine response after CLP-induced sepsis (mIL-1beta: p = 0.017; mIL-6: p < 0.0001; mTNF-α: p < 0.0001), preserved blood pressure control (p = 0.024) and down-regulated the binding activity of NF-κB. There were no changes in the pro-inflammatory cytokine response when peripheral blood was incubated with lipopolysaccharide alone compared with incubation with clonidine (10^-4 ^M) plus LPS (p > 0.05).

**Conclusions:**

Our results demonstrate that the pre-emptive administration of either clonidine or dexmedetomidine have the ability to successfully improve survival in experimental sepsis. Furthermore, there seems to be a connection between the central muscarinic network and the vagal cholinergic response. By down-regulating pro-inflammatory mediators sympatholytics may be a useful adjunct sedative in patients with a high risk for developing sepsis.

## Introduction

The use of the central acting alpha-2 agonist clonidine has become more popular since its introduction in the 1980s. Besides its classical use to treat hypertension [1], its area of use has spread further and is now used as an adjunct for epidural analgesia [2] and to treat withdrawal [3] or migraines [4]. It is also given as a pre-medication before cardiac surgery to treat cardiovascular fluctuations in high-risk patients [5]. Studies have shown that central acting alpha-2 agonists inhibit noradrenergic neurotransmission and have a strong sedative component secondary to sympathetic inhibition [6]. This formerly adverse side effect is nowadays widely used in critical care settings to sedate patients and to reduce the amount of co-medication needed. A recent study has shown the beneficial effects of dexmedetomidine over lorazepam as an adjunct sedative in a critical care setting [7]. Furthermore clonidine is an integral part of the sedation regimen in German intensive care units (ICU) [8].

The mechanism of action of central acting alpha-2 agonists like clonidine is based on their ability to reduce the central sympathetic tone by stimulating central alpha-2 receptors in the medulla oblongata [7,9,10]. Therefore, the balance shifts to the benefit of the parasympathetic nervous system. The exact pathway is not yet known, but recent evidence points towards the involvement of cerebral binding receptors for imidazol in the signal transduction cascade of clonidine [9]. The same mechanism of action is also true for dexmedetomidine, which is even more specific in acting as a central alpha-2 agonist [11].

Sepsis is one of the main causes of death in European and US ICUs. The onset and perpetuation of inflammation involves a complex network of cytokines such as TNF-α, IL-1β and IL-6, causing upregulation of controlling transcription factors such as nuclear factor-κB (NF-κB) [12,13]. Unbalanced overproduction of these mediators provokes overwhelming reactions resulting in systemic inflammation and eventually lethal multi-organ failure [14].

Evidence that the clinically used medication clonidine has the potential to be a prophylactic option in treating sepsis has come from Kim and colleagues [15]. They have shown that clonidine pre-medication is able to significantly reduce the pro-inflammatory cytokines IL-1β and IL-6 in patients undergoing hysterectomy. Another recent study has shown that clonidine used as an adjunct sedative in critically ill patients was able to reduce the occurrence of pneumonia in those patients [16].

We therefore hypothesise that the clinically used central alpha-2 agonist clonidine has the ability to improve survival in experimental sepsis by inhibiting the sympathetic tone and consequently inhibiting the release of the pro-inflammatory cytokine.

## Materials and methods

### Caecal ligation and puncture

Caecal ligation and puncture (CLP) was performed as described previously [17-20] after approval by the appropriate local review committee and in compliance with governmental guidelines. In brief, female C57BL/6 mice aged 12 to 16 weeks were anaesthetised by intraperitoneal administration of 100 mg/kg ketamine (Ketanest, Pfizer Pharma, Karlsruhe, Germany). The caecum was exposed through a 1.0 to 1.5 cm abdominal midline incision and subjected to a ligation 6 mm from the caecal tip followed by a single puncture with a G23 needle. A small amount of stool was expelled from the puncture to ensure patency. The caecum was returned into the peritoneal cavity and the abdominal incision was closed by layers with 5/0 prolene thread (Ethicon, Norderstedt, Germany). No antibiotics were administered in this model. For the sham-operated mice serving as controls, the caecum was mobilised but no ligation or puncture was performed.

Animals receiving pre-emptive injections were treated with either clonidine (5 μg/kg) or dexmedetomidine (40 μg/kg) 12 and 1 hours before the operation, as well as 1, 6 and 12 hours afterwards. Another group of animals only received clonidine (5 μg/kg) 1, 6 and 12 hours after the operation, while the pre-emptive injections were of normal saline. The control groups for each intervention received solvent (0.9% saline) injections at the corresponding time points. All injections were given intra-peritoneal. CLP was performed blinded to the identity of the treatment group. Survival after CLP was assessed four to six times a day for at least five days. Blood pressure was measured in anaesthetised mice with femoral manometric catheters 0, 6 and 24 hours after CLP.

To harvest blood for determination of cytokines, mice from the pre-emptive clonidine group and their corresponding controls were deeply anaesthetised by intraperitoneal injections of 30 μl/g Avertin (stock: 1 g tribromethanol/620 μl 2-methyl-1-butanol, 180 μl per 10 ml 0.9% sodium chloride (Braun, Melsungen, Germany)) 24 hours after CLP. To conduct electrophoretic mobility shift assay (EMSA) analysis and ELISA, the liver was immediately snap-frozen in liquid nitrogen.

### Electrophoretic mobility shift assay

Nuclear proteins from snap-frozen mouse tissues were isolated as described in detail elsewhere [21]. Nuclear extracts were assayed for NF-kB binding activity using the consensus sequence 5'-AGT TGA GGG GAC TTT CCC AGG C-3' labelled by [g-32P]-dATP and T4-kinase to a specific activity more than 5 × 10 cpm/μg. Specificity of binding was ascertained by competition with a 160-fold molar excess of unlabelled consensus oligonucleotides. Protein-DNA complexes were separated from unbound DNA probes by electrophoresis through 5% native polyacrylamide gels containing 2.5% glycerol and 0.5× tris-borate EDTA. Gels were dried and exposed to x-ray films (Amersham Pharmacia, Freiburg, Germany) at -80°C using intensifying screens for 48 to 60 hours.

### *In vitro *incubation of whole blood samples

The ethical principles as set out in the Declaration of Helsinki were honoured in the present study, which was approved by the ethics committee of the University of Heidelberg (S-123/2008). Venous blood was drawn from the antecubital vein of healthy volunteers after giving their informed consent. After discarding the first 2 mL, blood was collected in one 10th volume of citrate (3.8%, Becton Dickinson Vacutainer, New Jersey, USA) and samples were immediately used for the experiments. The samples were incubated for 24 hours either with lipopolysaccharide (1 μg) alone, with clonidine alone (10^-4 ^M) or with LPS (1 μg) plus clonidine (10^-4 ^M). In the latter experiments clonidine was added one hour before LPS administration. This was followed by centrifugation for 10 minutes at 2000 rpm, the serum was frozen at -80°C until ELISA was performed

### ELISA

Plasma and supernatants were subjected to ELISA for determination of Il-1β, Il-6 and TNF-α contents. ELISA kits were purchased from R&D Systems GmbH (Wiesbaden-Nordenstadt, Germany) and used according to the manufacturer's instructions.

### Statistical analysis

Where indicated, values of experimental groups are given as the mean, with bars showing the standard error of the mean (SEM). The means of the groups were compared by analysis of variance using the Student's t-test. When Kaplan-Meier estimates of group survival distributions are shown, the logrank test was used to compare two or more survival distributions. A result was denoted as statistically significant if the p-value of its corresponding test statistic was less than 0.05. All statistical computations were performed using Prism Graph (Version 5; GraphPad Software, La Jolla, CA., USA).

## Results

### Pre-emptive injection of the central acting alpha-2 agonist clonidine or dexmedetomidine improves survival in experimental sepsis

**Figure 1:**
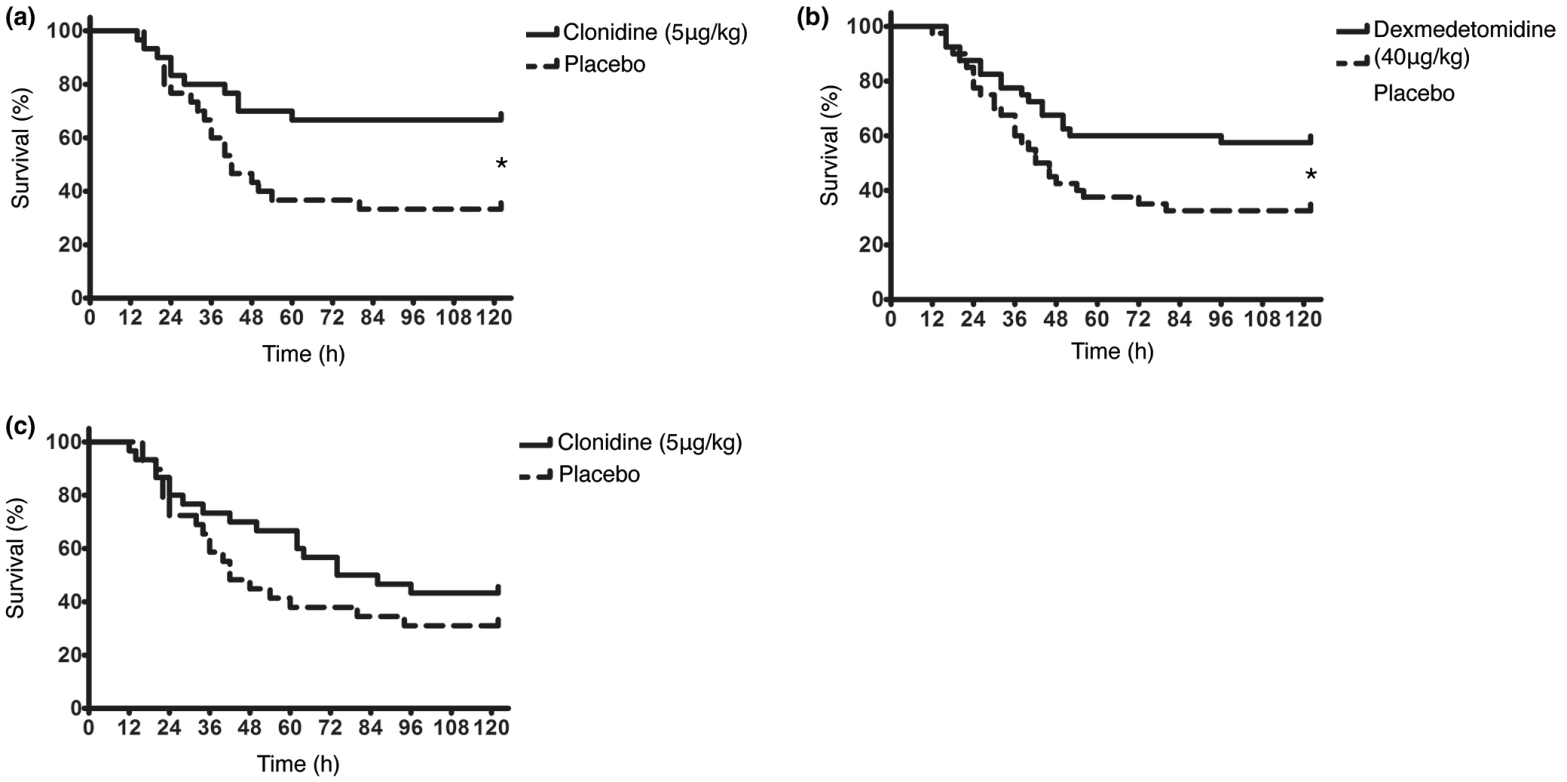
Survival after caecal ligation and puncture (CLP)-induced sepsis. Kaplan-Meier survival curves of mice after CLP-induced sepsis (t = 0). **(a)** Pre-emptive administration of clonidine improves survival after CLP-induced sepsis. The verum group (solid line, n = 30) received clonidine (5 μg/kg) 12 hours and 1 hour before CLP, as well as 1 hour, 6 hours and 12 hours after CLP. The control group received corresponding injections with solvent (dashed line, n = 30). Clonidine-treated animals survived significantly longer than their controls (p = 0.015, n = 60). **(b)** Pre-emptive administration of dexmedetomidine statistically prolongs survival after CLP-induced sepsis. The verum group (solid line, n = 40) received dexmedetomidine (40 μg/kg) 12 hours and 1 hour before CLP, as well as 1 hour, 6 hours and 12 hours after CLP. The control group received corresponding injections with solvent (dashed line, n = 40). Dexmedetomidine-treated animals survived significantly longer than their controls (p = 0.029, n = 80). **(c)** Post-operative administration of clonidine does not improve survival after CLP-induced sepsis. The verum group (solid line, n = 30) received clonidine (5 μg/kg) 1 hour, 6 hours and 12 hours after CLP. The control group received corresponding injections with solvent (dashed line, n = 30). There was no statistically significant difference between both groups (p = 0.228, n = 60). * indicates statistical significant differences between the groups.

The mice received a total of five injections of clonidine (5 μg/kg) intraperitoneally. The injections were given 12 hours and 1 hour before CLP and 1 hour, 6 hours and 12 hours after the intervention. The control group received solvent injections. Clonidine-treated animals survived significantly longer than control mice (p = 0.015; Figure 1). Notably, after survival of the initial phase, there were no further drop-outs within an observation period of five days, indicating a true resolution of sepsis rather than delaying kinetics.

We then tested if pre-emptive injection of the more specific central acting alpha-2 agonist dexmedetomidine would be sufficient to reduce mortality from sepsis. We therefore injected 40 μg/kg of dexmedetomidine 12 hours and 1 hour before and 1 hour, 6 hours and 12 hours after CLP and compared it with injection of solvent. Animals treated with dexmedetomidine survived significantly longer than the ones treated with solvent (p = 0.029; Figure 1).

We have shown above that pre-treatment with clonidine significantly improves survival in experimental sepsis if given pre-emptively before CLP. To further test if administration of clonidine after CLP would still be protective, we compared clonidine injections (5 μg/kg), given 1 hour, 6 hours and 12 hours after CLP, with solvent injections. The effect seen was much less pronounced compared with clonidine given before CLP. Mean survival times and Kaplan Meier survival curves showed no statistical difference (p = 0.228).

### Pre-emptive injection of clonidine reduces the generation of pro-inflammatory mediators, namely mIL-1β, mIL-6 and mTNF-α

**Figure 2:**
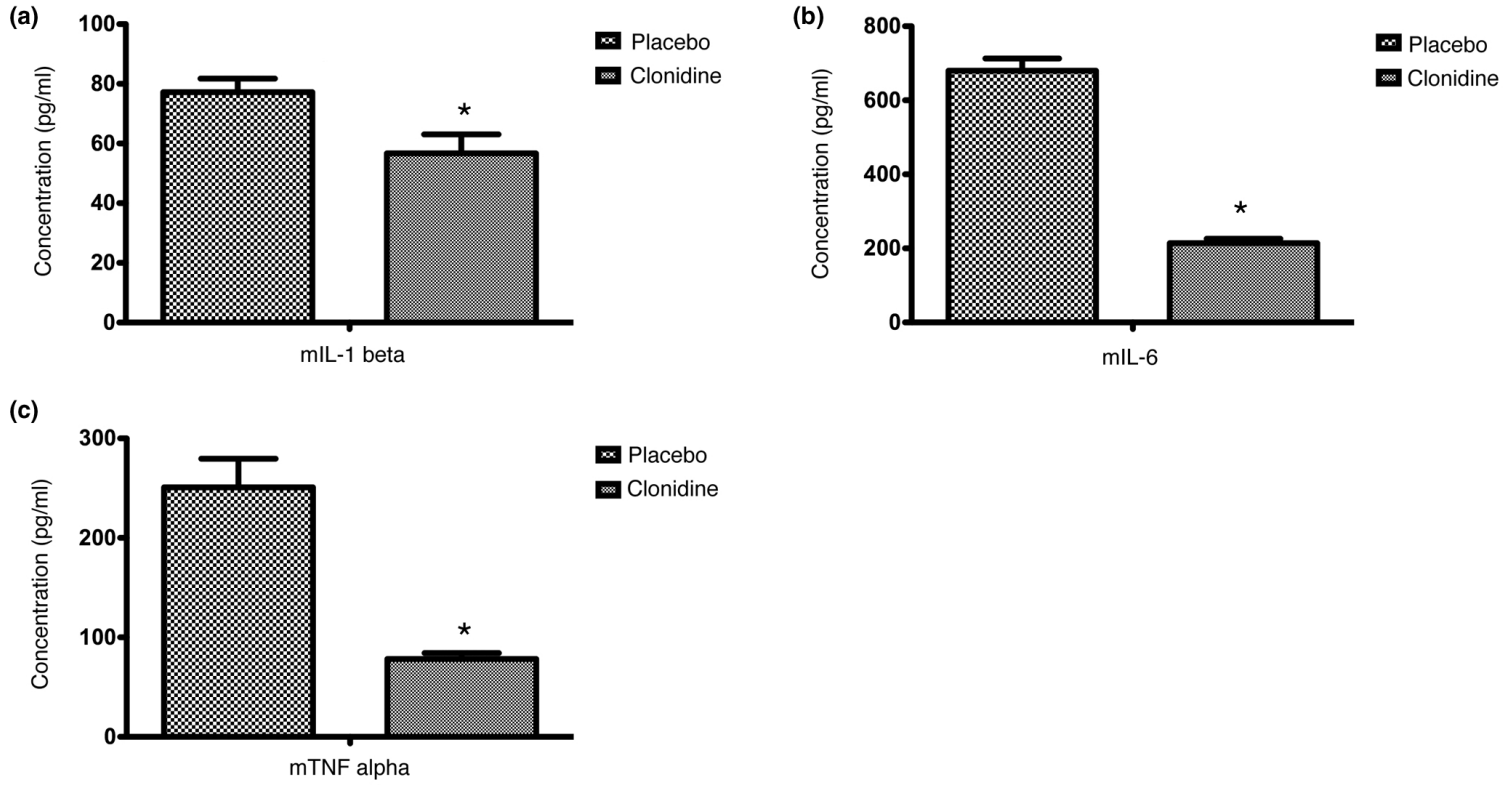
Clonidine attenuates cytokine response after caecal ligation and puncture (CLP)-induced sepsis. Mice in the verum group (n = 10) received clonidine (5 μg/kg) 12 hours and 1 hour before CLP, as well as 1 hour, 6 hours and 12 hours after CLP. The control group (n = 10) received injections with solvent respectively. **(a) **Pre-emptive administration of clonidine decreases mIL-1β levels after CLP induced sepsis. Experimental sepsis led to a profound increase in mIL-1β in mouse serum 24 hours after CLP. This increase was significantly attenuated by pre-emptive clonidine injections (p = 0.017) as described above. **(b)** Pre-emptive administration of clonidine decreases mIL-6 levels after CLP induced sepsis. Experimental sepsis led to a profound increase in mIL-6 in mouse serum 24 hours after CLP. This increase was significantly attenuated by pre-emptive clonidine injections (p < 0.0001) as described above. **(c)** Pre-emptive administration of clonidine decreases mTNF-α levels after CLP-induced sepsis. Experimental sepsis led to a profound increase in TNF-α in mouse serum 24 hours after CLP. This increase was significantly attenuated by pre-emptive clonidine injections (p < 0.0001) as described above. * indicates statistical significant differences between the groups.

We further investigated if the improved survival in the group pre-emptively treated with clonidine was correlated with decreased pro-inflammatory mediators. We elicited that experimental sepsis leads to a profound increase in mIL-1β in mouse serum 24 hours after CLP. This increase could be markedly attenuated by pre-emptive treatment with clonidine injections as described above (p = 0.017; Figure 2). The same effect could also be observed when mIL-6 was measured 24 hours after CLP. The increase initiated by sepsis was significantly reduced by pre-emptive clonidine injections (p < 0.0001; Figure 2). Furthermore the rise of the pro-inflammatory marker mTNF-α following CLP-induced sepsis was also significantly reduced by clonidine (p < 0.0001; Figure 2).

Those findings suggest that the decrease of the proinflammaty mediators mIL-6, mIL-1β and mTNF-α might contribute to the reduced mortality seen after clonidine injections.

### Pre-emptive injection of clonidine preserves blood pressure control in septic animals

To exclude that the effects of clonidine are attributed to the induction of hypotension we additionally monitored blood pressure in mice receiving pre-emptive administration of clonidine and compared it with mice only receiving normal saline (control group) at the corresponding time points (n = 12 for each group). At baseline (performance of CLP), blood pressures did not differ between both groups (114.0 ± 9.9 mmHg versus 109.7 ± 3.2 mmHg; p = 0.69). Furthermore, there was no difference between the two groups after six hours (97.0 ± 3.4 mmHg versus 96.1 ± 6.0 mmHg; p = 0.90) and there was no difference when compared with baseline (control group, baseline versus six hours: p = 0.08 and clonidine, baseline versus six hours: p = 0.10). However, after 24 hours mice receiving a pre-emptive administration of clonidine had relatively preserved blood pressures when compared with baseline (93.7 ± 4.6 mmHg versus 109.7 ± 3.2 mmHg; p = 0.02) while the control animals were significantly hypotensive when compared with baseline (114.0 ± 9.9 mmHg versus 76.4 ± 5.4 mmHg; p = 0.002) and also when compared with animals pre-emptively treated with clonidine (76.4 ± 5.4 mmHg versus 93.7 ± 4.6 mmHg; p = 0.024).

These results show that clonidine is not causing significant hypotension that contributes to its effect of prolonging survival or lowering cytokine response. In fact the pre-emptively treated animals have less hypotension caused by sepsis than the untreated ones, which points towards the protective effect of clonidine being independent of its ability to lower blood pressure.

### Pre-emptive injection of clonidine reduces NF-κB activation

**Figure 3:**
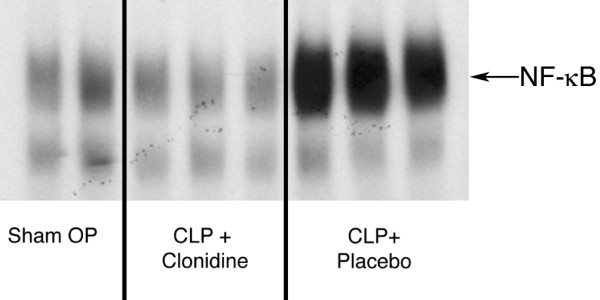
Clonidine reduces nuclear factor (NF) κB activation after caecal ligation and puncture (CLP)-induced sepsis. The figure shows NF-κB binding activity, as assessed by electrophoretic mobility shift assay (EMSA) assay in the livers of mice that underwent CLP surgery. CLP led to a strong increase in NF-κB binding activity. In contrast, in mice treated with clonidine we found no difference in NF-κB binding activity compared with mice receiving placebo injections with normal saline. Furthermore, there was no difference between mice with CLP-induced sepsis receiving clonidine and sham-operated mice. Group I 'Sham OP'; EMSA for NF-κB binding activity; liver tissue. Group II 'CLP + clonidine', clonidine therapy, EMSA for NF-κB binding activity; liver tissue. Group III: 'CLP + placebo', saline injection, EMSA for NF-κB binding activity; liver tissue

In addition to increased pro-inflammatory mediators measured in blood, CLP also leads to a strong increase in NF-κB binding activity assayed with EMSA in the liver (Figure 3). In contrast, in mice treated with clonidine we found no difference in NF-κB binding activity compared with mice receiving placebo injections with saline (Data not shown). Furthermore, there was no difference between mice with CLP-induced sepsis receiving clonidine and sham-operated animals (n = 6 for each group; Figure 3). This further contributes to the hypothesis that the survival improving effect of clonidine might be related to a decrease in pro-inflammatory mediators.

### Clonidine has no effect on cytokine production in LPS stimulated whole blood

**Figure 4:**
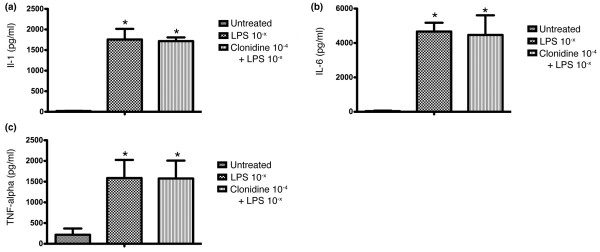
Whole blood incubation with lipopolysaccharide and clonidine respectively. Whole blood samples of healthy donors (n = 4) were incubated for 24 hours with either LPS alone (1 μg) or LPS (1 μg) plus clonidine (10^-4^). **(a) **IL-1β levels after whole blood incubation with LPS and clonidine respectively. The concentration of IL-1β increased significantly after LPS administration compared with untreated controls (p = 0.0005). There was no statistically significant difference in IL-1β levels between samples treated with LPS alone and those incubated with LPS plus clonidine (10^-4^; p = 0.89). **(b) **IL-6 levels after whole blood incubation with LPS and clonidine respectively. The concentration of IL-6 increased significantly after LPS administration compared with untreated controls (p = 0.0001). There was no statistically significant difference in IL-6 levels between samples treated with LPS alone and those incubated with LPS plus clonidine (10^-4^; p = 0.88). **(c) **TNF-α levels after whole blood incubation with LPS and clonidine respectively. The concentration of TNF-α increased significantly after LPS administration compared with untreated controls (p = 0.025). There was no statistically significant difference in TNF-α levels between samples treated with LPS alone and those incubated with LPS plus clonidine (10^-4^; p = 0.99). * indicates statistical significant differences when compared with untreated.

Next we tested if the effects described above were because of clonidine's central acting mechanism of action or mediated peripherally. Therefore, we incubated whole blood samples of healthy donors (n = 4) for 24 hours with either LPS (1 μg) alone, or clonidine (10^-4^) alone or LPS plus clonidine (10^-4^). As pro-inflammatory markers we choose IL-1β, IL-6 and TNF-α, as in the animal experiments. Administration of clonidine alone had no effect on the concentrations of either mediator chosen, compared with untreated controls (data not shown). Incubation with LPS on the other hand (100 μg/ml) showed a marked increase of each mediator compared with the untreated controls (LPS alone versus untreated control: p = 0.0005; Figure 4). This increase of IL-1β after LPS incubation did not significantly differ from incubation with LPS plus clonidine (LPS alone versus LPS plus clonidine 10^-4^: p = 0.89; Figure 4). The same was true for IL-6, which increased about the same amount after incubation with LPS alone or with LPS plus clonidine (LPS alone versus untreated control: p = 0.0001; LPS alone versus LPS plus clonidine 10^-4^: p = 0.88; Figure 4). Measurements of TNF-α also increased after the administration of LPS alone but this was not statistically different from administration of LPS and clonidine combined (LPS alone versus untreated control: p = 0.025; LPS alone versus LPS plus clonidine 10^-4^: p = 0.99; Figure 4).

The previously found data combined with the data above supports that the improved survival of CLP-induced sepsis in clonidine-treated animals is mediated centrally. This points to the importance of the balance between the sympathetic and parasympathetic nervous system in mediating an inflammatory response.

## Discussion

We have shown in this study that pre-emptive administration of clonidine or dexmedetomidine is able to significantly improve survival in sepsis induced by CLP. This is accompanied by a reduction in the pro-inflammatory mediators IL-1β, IL-6 and TNF-α, as well as a decrease in NF-kB binding activity, thereby inhibiting an overwhelming inflammation response to sepsis.

The use of central alpha-2 agonists as an adjunct sedative in critically ill patients in an ICU setting has been discussed in the scientific literature [7,8,11]. A first hint that sympatholytics are beneficial in sedating ICU patients comes from Pandharipande and colleagues who additionally refer to a vagomimetic component of dexmedetomidine [7]. They have shown that sedation with dexmedetomidine instead of lorazpam not only leads to significantly less delirum or coma, but also improves survival in those patients. Unfortunately, they did not report the incidence of sepsis in their patients. Another recent study, however, was able to show that patients treated with clonidine for alcohol withdrawl had significantly less pneumonia than those treated without a sympatholytic agent [16].

To determine the exact survival benefit in sepsis after sympathetic inhibition, we performed CLP, which causes lethal peritonitis by microbial infection and is a valid animal model for human sepsis [20,22]. Pre-emptive administration of either clonidine or dexmedetomidine was potent enough to significantly reduce mortality in CLP-induced sepsis. This supports the reasoning to consider the use of a sympatholytic drug as an adjunct sedative in both high-risk patients before undergoing major surgery and in ICU patients prone to sepsis. When the administration of clonidine was delayed until after the operation, no further protective effect could be elicited. This is consistent with previous studies that also failed to show a protective effect of late activation of the cholinergic anti-inflammatory pathway [23]. It contradicts the findings of Wang and colleagues [22] who demonstrated that nicotine treatment could be delayed for 24 hours after sepsis induction. This might be at least in part explained by the use of antibiotics in the study, whereas our animals did not receive any antibiotic treatment. Also different mice strains were used in that study which may be different in the susceptibility to CLP-induced peritonitis.

The improved survival in pre-emptively treated animals correlates with a reduction in NF-κB binding activity shown by EMSA. This corresponds to previous findings, showing a similar blunting of the NF-κB pathway on activation of the cholinergic anti-inflammatory pathway [22]. It is well known that most inflammatory signals merge in activation of the NF-κB pathway and NF-κB has been shown to play a critical role in modulating mortality in experimental [20] and clinical sepsis [24]. Thus, the elicited down-regulation of NF-κB binding activity after clonidine administration is a potential explanation for the improved survival in CLP-induced sepsis as shown here.

Furthermore pre-emptive administration of clonidine significantly reduced the pro-inflammatory mediators TNF-α, IL-6 and IL-1β, although these pro-inflammatory cytokines were still detectable in considerable concentrations. However, it has been shown that complete elimination of TNF-α after CLP-induced sepsis coincides with increased mortality [17,19]. Although increased levels of IL-6 seem to be predictive for poor survival and increased infectious complications [25]. Sympatholysis might therefore be sufficient to reduce cytokine levels low enough to prevent septic shock and consecutive death. It might also be necessary not to decrease cytokines too low in order to be sufficient for clearing bacterial infections.

Our study showed that peripheral *in vitro *administration of clonidine is not sufficient to reduce the pro-inflammatory cytokines measured. This finding supports the hypothesis that the beneficial effect of clonidine is not peripherally mediated but rather based on its ability to centrally activate alpha-2 receptors and thereby decrease sympathetic tone [6]. To date, the role of the balance between the sympathetic and parasympathetic nervous system has not been readily established. Recent studies, however, have suggested that there seems to be a connection between the central muscarinic network and the vagal cholinergic response [26], influencing each other. Furthermore, it has been shown that activation of the cholinergic anti-inflammatory pathway by vagal stimulation can be utilised to protect against experimental sepsis [22,23,27,28]. This was performed either directly by injection of nicotine [20], electrical stimulation of the vagal nerve [27] or indirectly by increasing the amount of acetylcholine (Ach) [23]. Our study points toward the possibility that direct sympatholysis might be sufficient enough to activate this pathway. It is interesting that the effect of clonidine does not correlate with significant hypotension that contributes to prolonging survival and lowering cytokine response. In fact the pre-emptively treated animals have improved blood pressure control and less hypotension than untreated ones.

Our study is limited by the use of ketamine as an anaesthetic for the CLP procedure. Ketamine is a noncompetitve inhibitor of the nicotinic Ach-receptor [29] that might leave the receptor unresponsive to Ach elevations induced by changes in vagal tone. However, in our study, animals received only a single dose of ketamine and the elimination half time of ketamine is about three hours. This might have attenuated the effects observed for the treatment with clonidine, which might have been even more pronounced if anaesthesia had been achieved using a different drug. Furthermore, there is also significant modulation of the Ach-haemostasis during surgical interventions.

Nevertheless, pre-emptive administration of either clonidine or dexmedetomidine was potent enough to significantly reduce mortality in CLP-induced sepsis. This supports the rationale to use clonidine or dexmedetomidine as an adjunct sedative in an ICU setting in order to reduce the occurrence of sepsis. Furthermore, administration of a central acting alpha-2 agonist might be considered as a pre-emptive therapeutic option in high-risk patients undergoing major surgery.

## Conclusions

We demonstrate that the clinically used central acting alpha-2 agonists clonidine and dexmedetomidine improve survival in murine experimental sepsis. This is most probably due to their sympatholytic effects that lead to down-regulation of pro-inflammatory mediators. Our findings provide a rationale for further exploration and may have important implications for the use of clonidine or dexmedetomidine as adjunct sedatives in the pre-emptive treatment of patients with a high risk for developing sepsis.

## Key messages

• The clinically used central acting alpha-2 agonists clonidine and dexmedetomidene improve survival in murine experimental sepsis

• Down-regulation of pro-inflammatory mediators due to sympatholytic effects of above mentioned drugs most probably responsible for this effect

• Sympatholytics like clonidine or dexmedetomidine may therefore be useful adjunct sedatives in the pre-emptive treatment of patients with a high risk for developing sepsis

## Abbreviations

Ach: acetylcholine; CLP: caecal ligation and puncture; ELISA: enzyme immunosorbent assay; EMSA: electrophoretic mobility shift assay; ICU: intensive care unit; IL: interleukin; NF: nuclear factor; SEM: standard error of the mean; TNF: tumour necrosis factor.

## Competing interests

The authors declare that they have no competing interests.

## Authors' contributions

SH, JS, TW, MW and EM designed the study. SH and TW acquired the data. JS and TW analysed the data. SH, JS, MW, BMG, AB, BF and CL wrote the manuscript.
